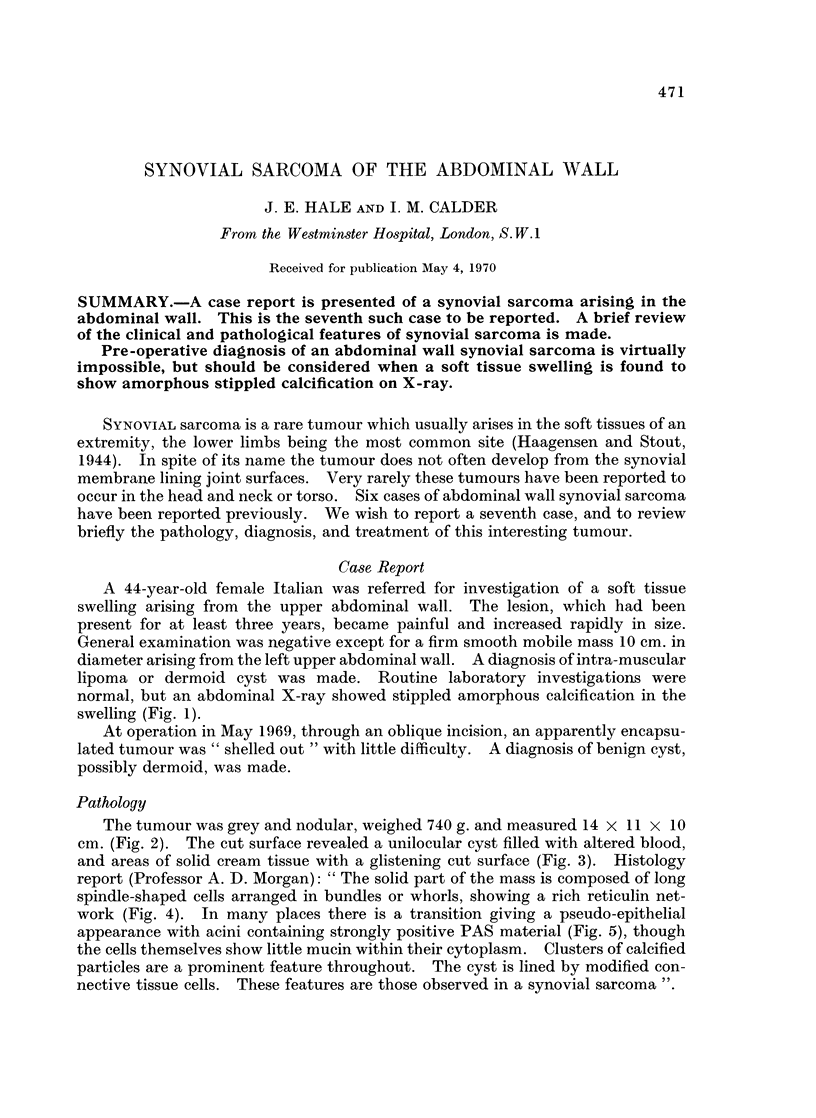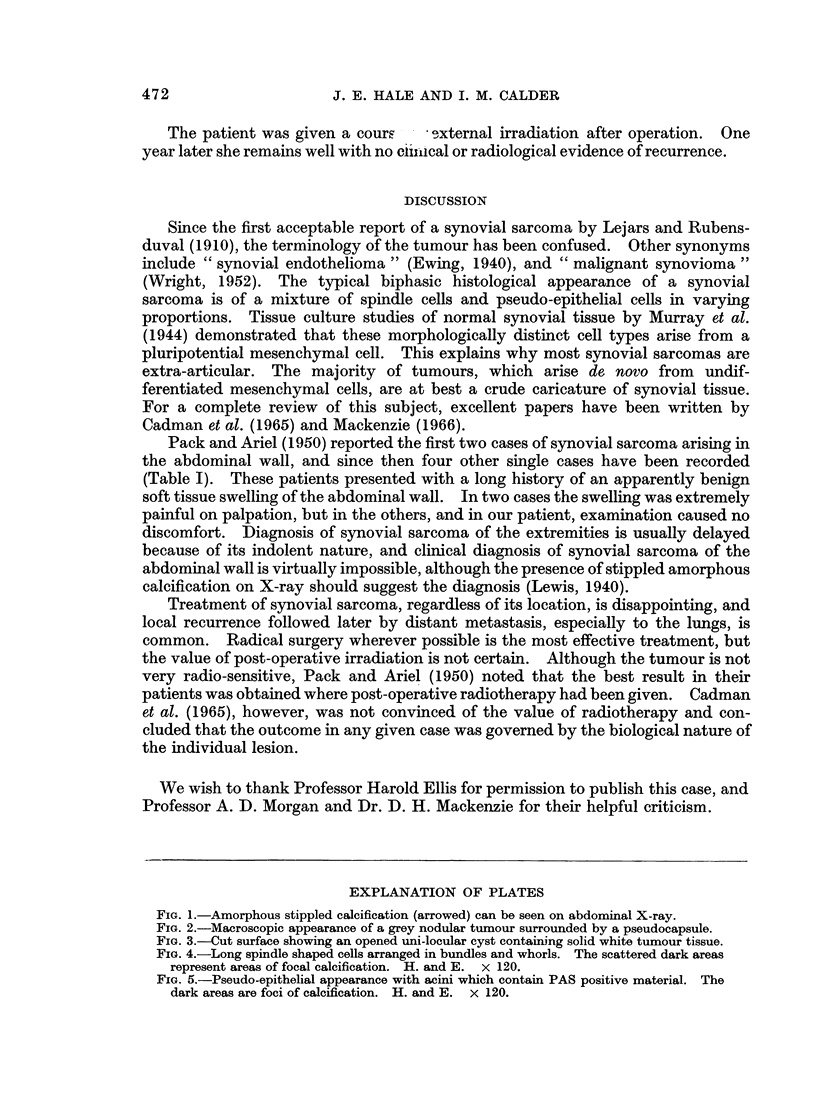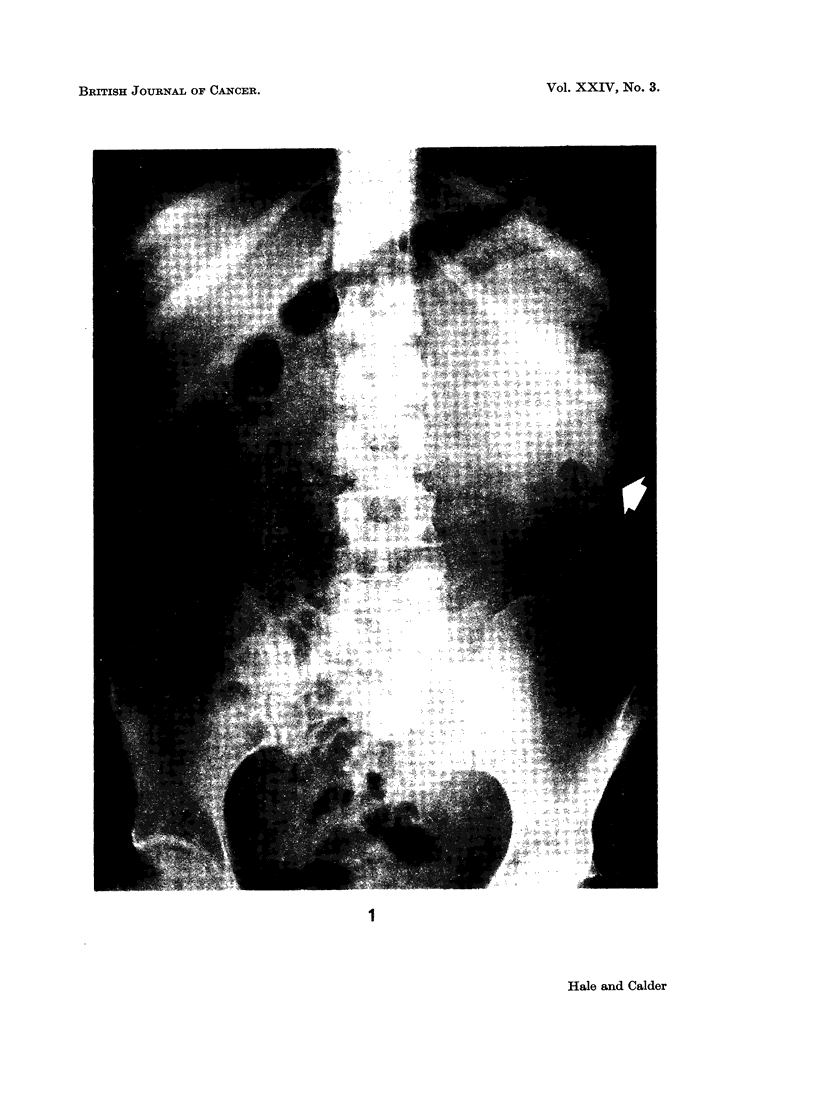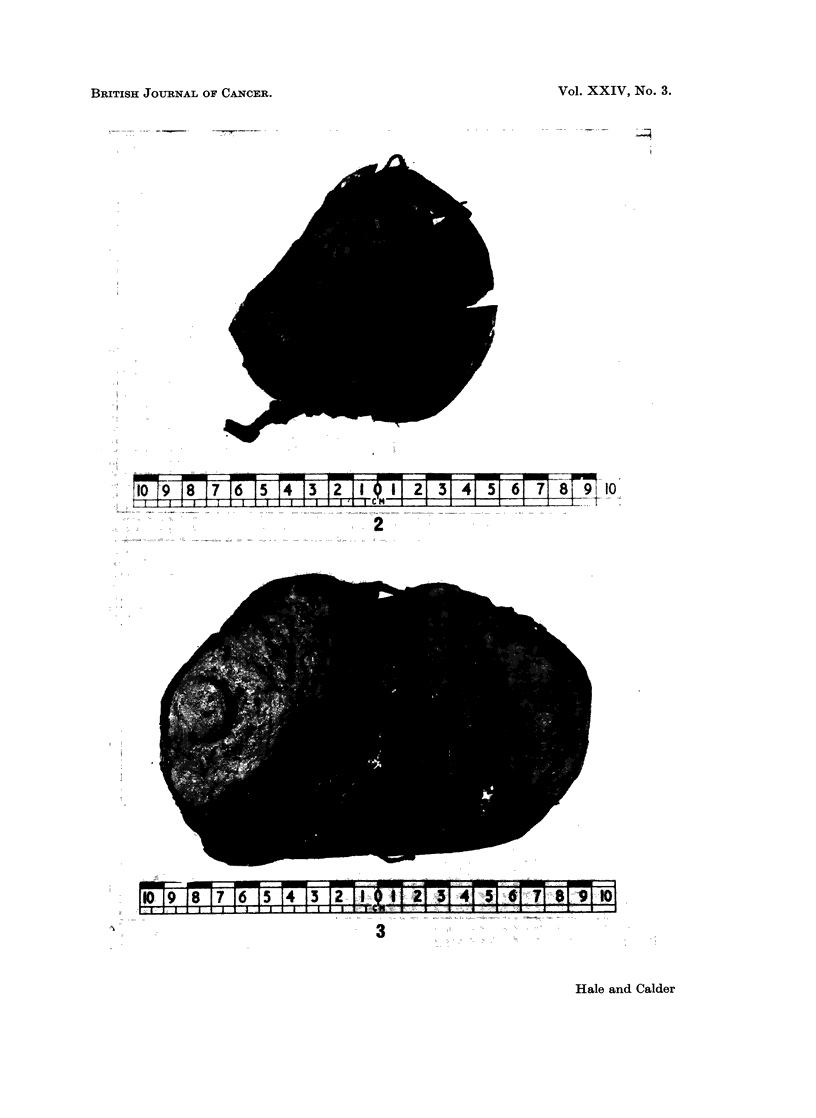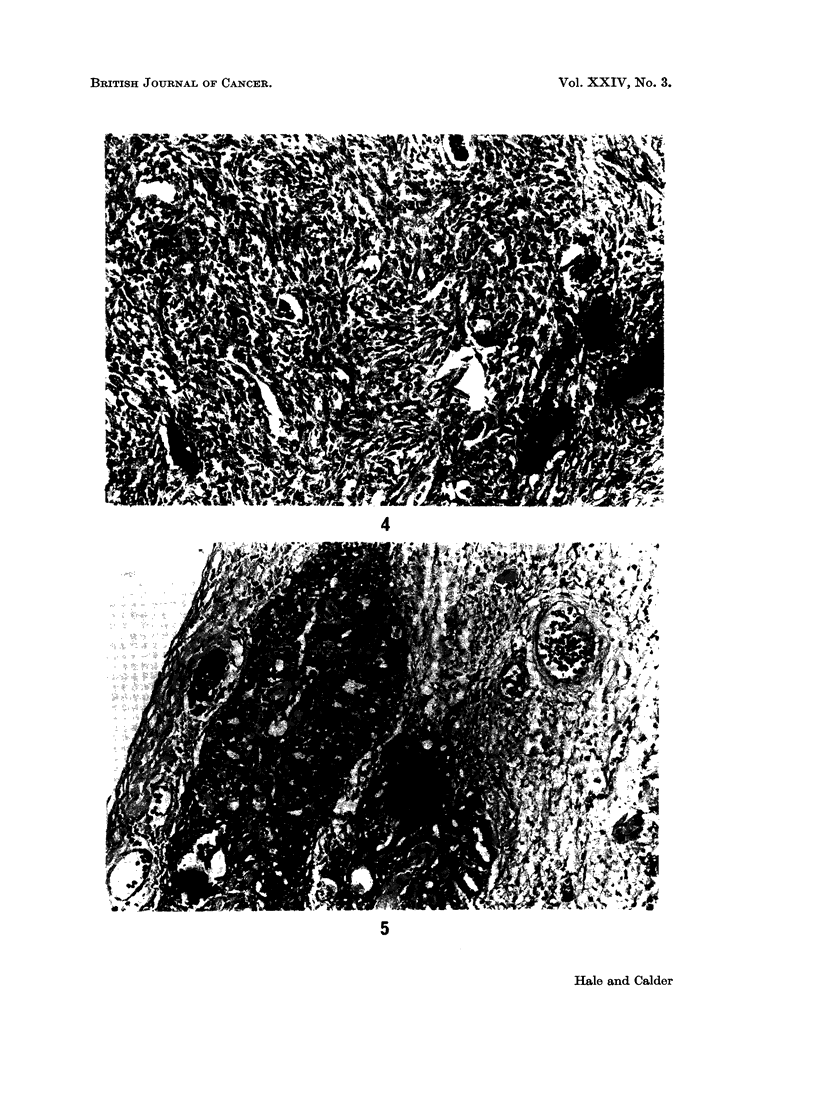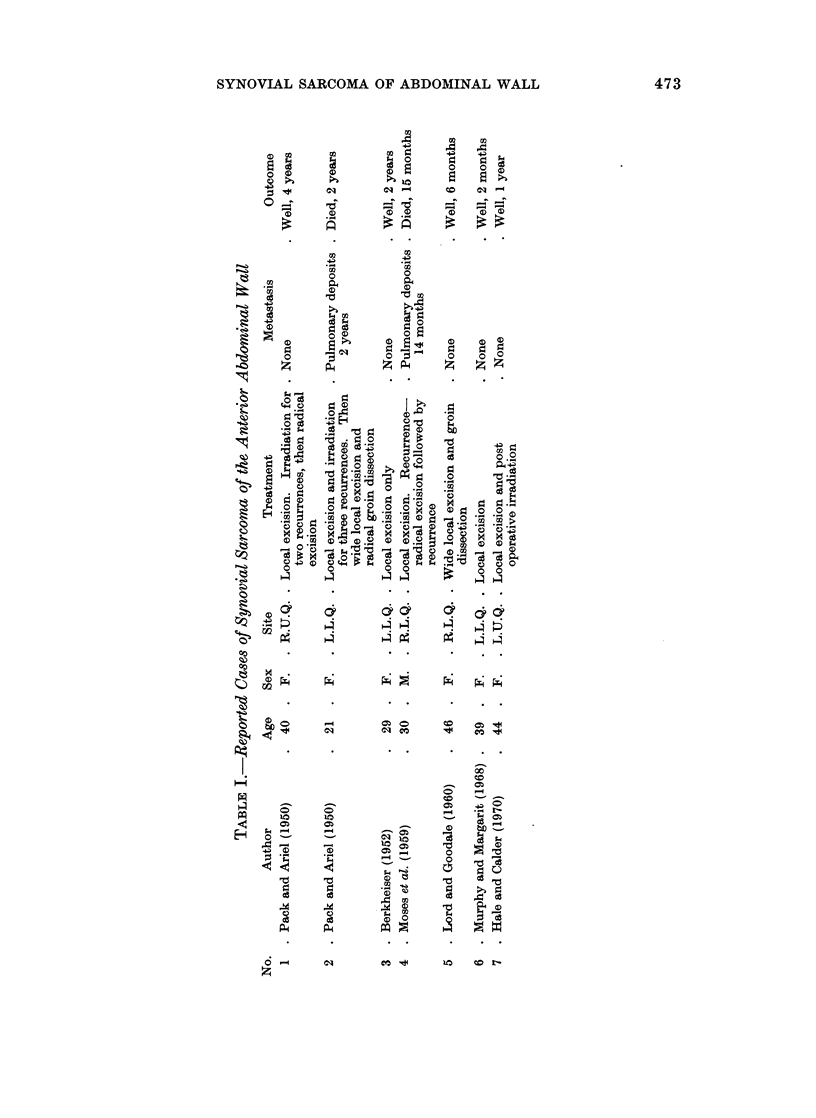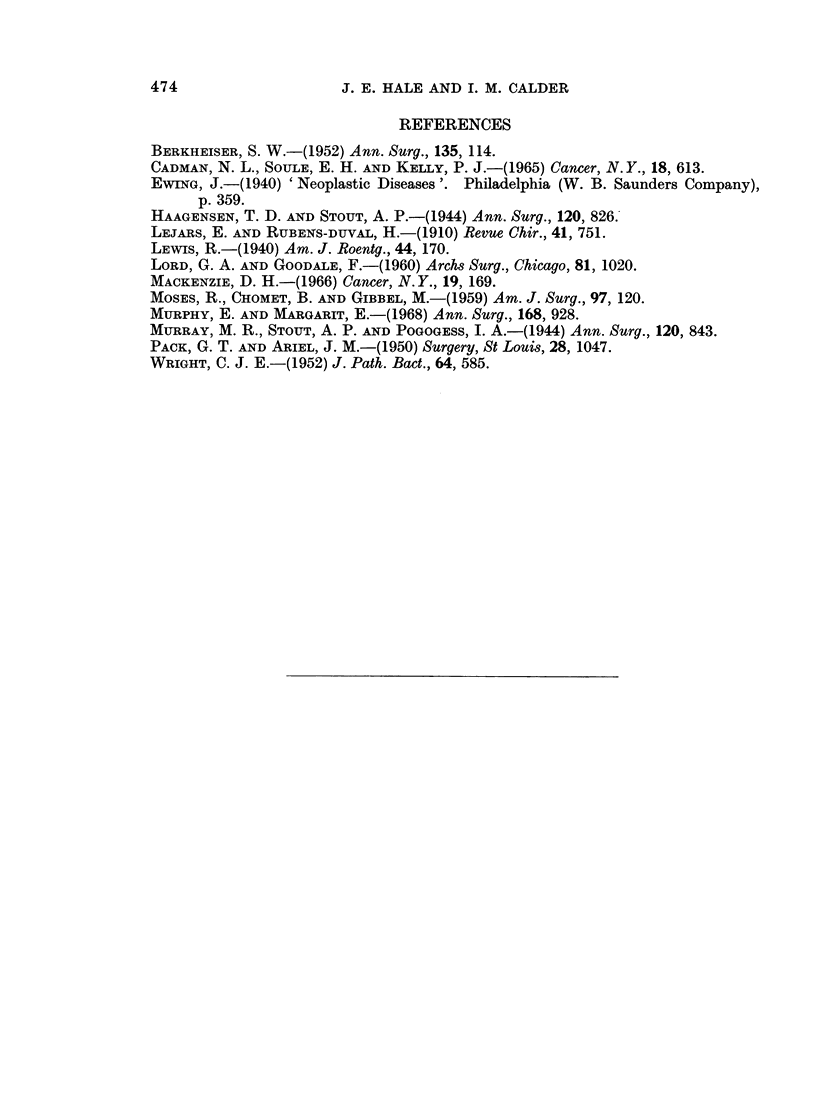# Synovial Sarcoma of the Abdominal Wall

**DOI:** 10.1038/bjc.1970.56

**Published:** 1970-09

**Authors:** J. E. Hale, I. M. Calder

## Abstract

**Images:**


### SYNOVIAL SARCOMA OF THE ABDOMINAL WALL

J. E. HALE AND I. M. CALDER

*From the Westminster Hospital, London, S.W.1*

Received for publication May 4, 1970

**SUMMARY.—A case report is presented of a synovial sarcoma arising in the abdominal wall. This is the seventh such case to be reported. A brief review of the clinical and pathological features of synovial sarcoma is made.**

**Pre-operative diagnosis of an abdominal wall synovial sarcoma is virtually impossible, but should be considered when a soft tissue swelling is found to show amorphous stippled calcification on X-ray.**

SYNOVIAL sarcoma is a rare tumour which usually arises in the soft tissues of an extremity, the lower limbs being the most common site (Haagensen and Stout, 1944). In spite of its name the tumour does not often develop from the synovial membrane lining joint surfaces. Very rarely these tumours have been reported to occur in the head and neck or torso. Six cases of abdominal wall synovial sarcoma have been reported previously. We wish to report a seventh case, and to review briefly the pathology, diagnosis, and treatment of this interesting tumour.

### *Case Report*

A 44-year-old female Italian was referred for investigation of a soft tissue swelling arising from the upper abdominal wall. The lesion, which had been present for at least three years, became painful and increased rapidly in size. General examination was negative except for a firm smooth mobile mass 10 cm. in diameter arising from the left upper abdominal wall. A diagnosis of intra-muscular lipoma or dermoid cyst was made. Routine laboratory investigations were normal, but an abdominal X-ray showed stippled amorphous calcification in the swelling (Fig. 1).

At operation in May 1969, through an oblique incision, an apparently encapsulated tumour was " shelled out " with little difficulty. A diagnosis of benign cyst, possibly dermoid, was made.

### *Pathology*

The tumour was grey and nodular, weighed 740 g. and measured 14 $\times$ 11 $\times$ 10 cm. (Fig. 2). The cut surface revealed a unilocular cyst filled with altered blood, and areas of solid cream tissue with a glistening cut surface (Fig. 3). Histology report (Professor A. D. Morgan): " The solid part of the mass is composed of long spindle-shaped cells arranged in bundles or whorls, showing a rich reticulin network (Fig. 4). In many places there is a transition giving a pseudo-epithelial appearance with acini containing strongly positive PAS material (Fig. 5), though the cells themselves show little mucin within their cytoplasm. Clusters of calcified particles are a prominent feature throughout. The cyst is lined by modified connective tissue cells. These features are those observed in a synovial sarcoma ".

The patient was given a cours[illegible] external irradiation after operation. One year later she remains well with no clinical or radiological evidence of recurrence.

### DISCUSSION

Since the first acceptable report of a synovial sarcoma by Lejars and Rubens-duval (1910), the terminology of the tumour has been confused. Other synonyms include " synovial endothelioma " (Ewing, 1940), and " malignant synovioma " (Wright, 1952). The typical biphasic histological appearance of a synovial sarcoma is of a mixture of spindle cells and pseudo-epithelial cells in varying proportions. Tissue culture studies of normal synovial tissue by Murray *et al.* (1944) demonstrated that these morphologically distinct cell types arise from a pluripotential mesenchymal cell. This explains why most synovial sarcomas are extra-articular. The majority of tumours, which arise *de novo* from undifferentiated mesenchymal cells, are at best a crude caricature of synovial tissue. For a complete review of this subject, excellent papers have been written by Cadman *et al.* (1965) and Mackenzie (1966).

Pack and Ariel (1950) reported the first two cases of synovial sarcoma arising in the abdominal wall, and since then four other single cases have been recorded (Table I). These patients presented with a long history of an apparently benign soft tissue swelling of the abdominal wall. In two cases the swelling was extremely painful on palpation, but in the others, and in our patient, examination caused no discomfort. Diagnosis of synovial sarcoma of the extremities is usually delayed because of its indolent nature, and clinical diagnosis of synovial sarcoma of the abdominal wall is virtually impossible, although the presence of stippled amorphous calcification on X-ray should suggest the diagnosis (Lewis, 1940).

Treatment of synovial sarcoma, regardless of its location, is disappointing, and local recurrence followed later by distant metastasis, especially to the lungs, is common. Radical surgery wherever possible is the most effective treatment, but the value of post-operative irradiation is not certain. Although the tumour is not very radio-sensitive, Pack and Ariel (1950) noted that the best result in their patients was obtained where post-operative radiotherapy had been given. Cadman *et al.* (1965), however, was not convinced of the value of radiotherapy and concluded that the outcome in any given case was governed by the biological nature of the individual lesion.

We wish to thank Professor Harold Ellis for permission to publish this case, and Professor A. D. Morgan and Dr. D. H. Mackenzie for their helpful criticism.

---

### EXPLANATION OF PLATES

FIG. 1.—Amorphous stippled calcification (arrowed) can be seen on abdominal X-ray.
FIG. 2.—Macroscopic appearance of a grey nodular tumour surrounded by a pseudocapsule.
FIG. 3.—Cut surface showing an opened uni-locular cyst containing solid white tumour tissue.
FIG. 4.—Long spindle shaped cells arranged in bundles and whorls. The scattered dark areas represent areas of focal calcification. H. and E. × 120.
FIG. 5.—Pseudo-epithelial appearance with acini which contain PAS positive material. The dark areas are foci of calcification. H. and E. × 120.

1

Hale and Calder

2

3

Hale and Calder

4

5

Hale and Calder

TABLE I.—*Reported Cases of Synovial Sarcoma of the Anterior Abdominal Wall*

| No. | Author | Age | Sex | Site | Treatment | Metastasis | Outcome |
|---|---|---|---|---|---|---|---|
| 1 | Pack and Ariel (1950) | 40 | F. | R.U.Q. | Local excision. Irradiation for two recurrences, then radical excision | None | Well, 4 years |
| 2 | Pack and Ariel (1950) | 21 | F. | L.L.Q. | Local excision and irradiation for three recurrences. Then wide local excision and radical groin dissection | Pulmonary deposits 2 years | Died, 2 years |
| 3 | Berkheiser (1952) | 29 | F. | L.L.Q. | Local excision only | None | Well, 2 years |
| 4 | Moses *et al.* (1959) | 30 | M. | R.L.Q. | Local excision. Recurrence—radical excision followed by recurrence | Pulmonary deposits 14 months | Died, 15 months |
| 5 | Lord and Goodale (1960) | 46 | F. | R.L.Q. | Wide local excision and groin dissection | None | Well, 6 months |
| 6 | Murphy and Margarit (1968) | 39 | F. | L.L.Q. | Local excision | None | Well, 2 months |
| 7 | Hale and Calder (1970) | 44 | F. | L.U.Q. | Local excision and post operative irradiation | None | Well, 1 year |

### REFERENCES

Berkheiser, S. W.—(1952) *Ann. Surg.*, **135**, 114.
Cadman, N. L., Soule, E. H. and Kelly, P. J.—(1965) *Cancer, N.Y.*, **18**, 613.
Ewing, J.—(1940) 'Neoplastic Diseases'. Philadelphia (W. B. Saunders Company), p. 359.
Haagensen, T. D. and Stout, A. P.—(1944) *Ann. Surg.*, **120**, 826.
Lejars, E. and Rubens-duval, H.—(1910) *Revue Chir.*, **41**, 751.
Lewis, R.—(1940) *Am. J. Roentg.*, **44**, 170.
Lord, G. A. and Goodale, F.—(1960) *Archs Surg., Chicago*, **81**, 1020.
Mackenzie, D. H.—(1966) *Cancer, N.Y.*, **19**, 169.
Moses, R., Chomet, B. and Gibbel, M.—(1959) *Am. J. Surg.*, **97**, 120.
Murphy, E. and Margarit, E.—(1968) *Ann. Surg.*, **168**, 928.
Murray, M. R., Stout, A. P. and Pogogess, I. A.—(1944) *Ann. Surg.*, **120**, 843.
Pack, G. T. and Ariel, J. M.—(1950) *Surgery, St Louis*, **28**, 1047.
Wright, C. J. E.—(1952) *J. Path. Bact.*, **64**, 585.